# Supplementary material for: Ten Considerations for Integrating Patient-Reported Outcomes into Clinical Care for Childhood Cancer Survivors
Source: Cancers (Basel). 2023 Feb 6;15(4):1024. doi: 10.3390/cancers15041024 (PMC9954048; doi:10.3390/cancers15041024)
Supplement: Supplementary file 1 [file cancers-15-01024-s001.zip › cancers-2174561-supplementary.pdf]

Supplementary Materials

Table S1: Measurement properties: Definitions and required evidence

Measurement property	Definition	Evidence required
Content validity	The degree to which the content of the PRO instrument is an adequate reflection of the construct to be measured.	• Subjective judgment by stakeholders (patients, survivors, caregivers, healthcare professionals and PRO content experts) through semi-structured interview, focus group discussion or survey
Internal consistency	The degree of the interrelatedness among the items.	• Cronbach's alpha coefficient<br>• Omega coefficient<br>• Item-total correlation coefficient
Reliability (test-retest or inter-rater)	The proportion of the total variance in the measurements which is because of "true" differences among patients. A person's "true" score is the average score that would be obtained if the scale was administered an infinite number of times to the same person.	• Kappa coefficient<br>• Intraclass correlation coefficient (ICC)
Measurement error	The systematic and random error of a patient's score that is not attributed to true changes in the construct to be measured.	• Standard error of measurement (SEM)<br>• Smallest detectable change (SDC)<br>• Limits of agreement (LoA)<br>• Percentage agreement (for nominal/ordinal measures)
Construct validity/known-group validity	The degree to which the scores of a PRO instrument are consistent with hypotheses (for instance with regard to internal relationships, relationships to scores of other instruments, or differences between relevant groups) based on the assumption that the PRO instrument validly measures the construct to be measured.	• Correlation between the instrument and another measure<br>• Association/difference between two groups hypothesized to have different levels of the construct of interest (e.g., pediatric cancer patients vs. healthy siblings, high vs. low intensity of cancer treatment)
Structural validity	The degree to which the scores of a PRO instrument are an adequate reflection of the dimensionality of the construct to be measured.	• Factor analysis<br>• Item response theory (IRT)<br>• Rasch analysis

Cross-cultural validity/ measurement invariance	Cross-cultural validity is the degree to which the performance of the items on a translated or culturally adapted PRO instrument is an adequate reflection of the performance of the items of the original version of the PRO instrument.<br><br>Measurement invariance is, in a way, the inverse of cross-cultural validity and is the ability of a PRO instrument to measure the latent construct similarly across different groups. Measurement invariance differences in scores can be attributed to differences in the latent construct, not measurement differences.	• Differential item functioning test (DIF)<br>• Multi-group confirmatory factor analysis (MGCFA)
Criterion validity	The degree to which the scores of a PRO instrument are an adequate reflection of a "gold standard."	• Correlation between a target measure and a gold standard measure
Responsiveness to change	The ability of a PRO instrument to detect change over time in the construct to be measured.	• Challenging hypotheses about the amount and direction of expected change<br>• Anchor-based methods: area under the ROC curve (AUC)<br>• Distribution-based methods: effect sizes<br>• Note: Guyatt's responsiveness ratio and paired t-tests are inappropriate measures of responsiveness.
Interpretability	The degree to which one can assign qualitative meaning – that is, clinical or commonly understood connotations – to an instrument's quantitative scores or change in scores.	• For single scores: distribution of scores<br>• For change scores: MIDs
Feasibility	The ease of application of the PRO instrument in its intended context of use, given constraints such as time or money.	• Completion time<br>• Cost<br>• Length of the instrument<br>• Type and ease of administration
Predictive validity	Demonstration of the relationship between selection procedure scores and some future work behavior or work outcomes.	• Correlation between PRO scores and subsequent assessment of a different health outcomes
Cut points/Minimal important differences	A cutoff score is a score at or above which applicants are selected for further consideration in the selection	For cut points:<br>• Area under the ROC curve<br>• Sensitivity

	process. Cutoff scores are not necessarily criterion-referenced, and different organizations may establish different cutoff scores on the same selection procedure based on their needs.<br><br>The minimal important difference (MID) is the smallest difference in score in the domain of interest which patients perceive as beneficial and which would mandate, in the absence of troublesome side effects and excessive cost, a change in the patient's management.	• Specificity<br><br>For MIDs:<br>• Anchor-based methods: area under the ROC curve (AUC)<br>• Distribution-based methods: effect sizes<br>• Scale-judgment methods
Response shift	A change in the meaning of one's self-evaluation of a target construct as a result of: (a) a change in the respondent's internal standards of measurement (i.e., scale recalibration); (b) a change in the respondent's values (i.e., the importance of component domains constituting the target construct) or (c) a redefinition of the target construct (i.e., reconceptualization).	• Repertory Grid Technique<br>• Cantril's Ladder<br>• Schedule for the Evaluation of Individual Quality of Life (SEIQoL)<br>• Extended Q-TWiST<br>• Preference mapping<br>• Card sorting<br>• Then-Test<br>• Ideal scale approach<br>• Structural equation modeling<br>• Growth curve analysis<br>• Idiographic assessment of personal goals<br>• Cognitive appraisal
Score calculation	In clinical research, mean or sum scores are most often used. In psychometric theory, more rigorous methods are available to calculate a score such as factor scores, regression scores, or IRT-derived scores.	• Mean scores<br>• Sum scores<br>• Factor analysis<br>• IRT

Note. Definitions from the COSMIN guidelines (Mokkink, et al., J Clin Epidemiol 2010 [5]; Prinsen, et al., Qual Life Res 2018 [6]; See references listed below for details) and previously published definitions (Tippins, et al., Ind Organ Psychol 2018 [9]; Chung, et al., Qual Life Res 2014 [1]; Jaeschke, et al., Control Clin Trials 1989 [4]; Schwartz, et al., Soc Sci Med 1999 [7]; Feißt, et al., BMC Med Res Methodol 2019 [2]; Hays, et al., Med Care 2000 [3]; Stover, et al., J Patient Rep Outcomes 2019 [8]; See references listed below for details)

References

1. Chung, H.; Kim, J.; Cook, K.F.; Askew, R.L.; Revicki, D.A.; Amtmann, D. Testing measurement invariance of the patient-reported outcomes measurement information system pain behaviors score between the US general population sample and a sample of individuals with chronic pain. *Qual Life Res* **2014**, *23*, 239-244, doi:10.1007/s11136-013-0463-0.
2. Feißt, M.; Hennigs, A.; Heil, J.; Moosbrugger, H.; Kelava, A.; Stolpner, I.; Kieser, M.; Rauch, G. Refining scores based on patient reported outcomes – statistical and medical perspectives. *BMC Med Res Methodol* **2019**, *19*, doi:10.1186/s12874-019-0806-9.
3. Hays, R.D.; Morales, L.S.; Reise, S.P. Item Response Theory and health outcomes measurement in the 21st century. *Med Care* **2000**, *38*, II-28-II-42, doi:10.1097/00005650-200009002-00007.
4. Jaeschke, R.; Singer, J.; Guyatt, G.H. Measurement of health status. Ascertaining the minimal clinically important difference. *Control Clin Trials* **1989**, *10*, 407-415, doi:10.1016/0197-2456(89)90005-6.
5. Mokkink, L.B.; Terwee, C.B.; Patrick, D.L.; Alonso, J.; Stratford, P.W.; Knol, D.L.; Bouter, L.M.; De Vet, H.C.W. The COSMIN study reached international consensus on taxonomy, terminology, and definitions of measurement properties for health-related patient-reported outcomes. *J Clin Epidemiol* **2010**, *63*, 737-745, doi:10.1016/j.jclinepi.2010.02.006.
6. Prinsen, C.A.C.; Mokkink, L.B.; Bouter, L.M.; Alonso, J.; Patrick, D.L.; De Vet, H.C.W.; Terwee, C.B. COSMIN guideline for systematic reviews of patient-

reported outcome measures. *Qual Life Res* **2018**, *27*, 1147-1157, doi:10.1007/s11136-018-1798-3.

7. Schwartz, C.E.S., M.A.G. Methodological approaches for assessing response shift in longitudinal health-related quality-of-life research. *Soc Sci Med* **1999**, *48*, 1531-1548, doi:10.1016/s0277-9536(99)00047-7.
8. Stover, A.M.; McLeod, L.D.; Langer, M.M.; Chen, W.-H.; Reeve, B.B. State of the psychometric methods: Patient-reported outcome measure development and refinement using item response theory. *J Patient-Rep Outcomes* **2019**, *3*, doi:10.1186/s41687-019-0130-5.
9. Tippins, N.; Sackett, P.; Oswald, F. Principles for the validation and use of personnel selection procedures. *Ind Organ Psychol* **2018**, *11*, 1-97.
